# Femoroacetabular impingement: question-driven review of hip joint pathophysiology from asymptomatic skeletal deformity to end-stage osteoarthritis

**DOI:** 10.1186/s10195-019-0539-x

**Published:** 2019-11-04

**Authors:** L. Pierannunzii

**Affiliations:** Orthopaedic Surgeon, ASST Gaetano Pini-CTO, Piazza Cardinale Andrea Ferrari, 1, 20122 Milan, Italy

**Keywords:** FAI, Femoroacetabular impingement, Contact mechanics, Shear stress, Normal stress, Inflammation, Labrum, Hip osteoarthritis

## Abstract

**Abstract:**

Femoroacetabular impingement (FAI), together with its two main pathomechanisms, cam and pincer, has become a trending topic since the end of the 1990s. Despite massive academic research, this hip disorder still conceals obscure aspects and unanswered questions that only a question-driven approach may settle. The pathway that leads a FAI asymptomatic morphology through a FAI syndrome to a FAI-related osteoarthritis is little known. Contact mechanics provides a shareable and persuasive perspective: cam FAI is based on shear contact stress at joint level with consequent cartilage wear; pincer FAI, contrariwise, determines normal contact stress between acetabular rim and femoral neck and squeezes the labrum in between, with no cartilage wear for many years from the onset. Pincer prognosis is then far better than cam. As a matter of fact, cartilage wear releases fragments of extracellular matrix which in turn trigger joint inflammation, with consequently worsening lubrication and further enhanced wear. Inflammation pathobiology feeds pathotribology through a vicious loop, finally leading to hip osteoarthritis. The association of cam and pincer, possibly overdiagnosed, is a synergic combination that may damage the joint rapidly and severely. The expectations after FAI surgical correction depend strictly on chondral layer imaging, on time elapsed from the onset of symptoms and on clinic-functional preoperative level. However, preemptive surgical correction is not recommended yet in asymptomatic FAI morphology.

**Level of evidence:**

V.

## Introduction

Since the end of the 1990s, when Reinhold Ganz and coworkers published their preliminary observations about a rare complication of periacetabular osteotomy due to hypercorrection of the dysplastic acetabulum or underestimation of the associated lack of antero-lateral head–neck offset [1], the academic research on femoro-acetabular impingement (FAI) has been generous, methodologically sound, inspired by brilliant intuitions and based on prolific interdisciplinary collaboration. Using PubMed/MEDLINE, and searching for “cam AND hip” 975 articles are retrieved, while searching for “pincer” AND “hip”, another 380 are found. Lastly, when we look for “FAI,” we find 2306 items, as of December 2018. Undoubtedly, some of these results do not match our query, especially if the search is extended to before 2000, but still the numbers are outstanding for a recently acknowledged single-joint disease. Despite all these academic efforts, the basic mechanisms of articular FAI (Table 1) still conceal gray areas and unanswered questions (e.g., which morphologic variables should be considered diagnostic and with what thresholds? Why is FAI morphology often symptom-free? Do cam and pincer have a similar prognosis? Does surgical correction of FAI morphology prevent the hip from becoming painful or osteoarthritic?…). These considerations justify further reviewing a subject probably already over-reviewed, but almost surely not exhaustively. Question-driven thinking is a valuable methodology used in many fields of Social Sciences, both in teaching and in research, but recently it might have been too often ignored in medical investigations, at least in this field of Orthopedics. The latest developments in computer science made it feasible to collect, store, analyze and share large sets of biomedical data that might have diverted our attention from the “questions that really matter” for clinicians to the “advanced methods you cannot do without (if you aim to publish your study in a prestigious journal)” for statisticians. Following on from big data, most recent publications about FAI could be dangerously confusing for the unprepared reader, who is more familiar with question-driven research than with data- or method-driven ones. The social scientist Davidson titled one of her articles [2] “*How we limit what we learn by limiting what we ask*”; and this is perfectly illustrated in many recent papers in the above 2306. The purpose of this review is to organize, in a narrative way, the evidence collected till now in order to answer the questions that really matter about morphology, clinical presentation, pathomechanics, management options and prognosis of this condition.

**Table 1:** Cam and pincer, similarities and differences

	Cam	Pincer
Common location	Femoral head–neck junction	Acetabular rim
Primary damage	Acetabular cartilage wear	Labrum squeezing
Prevailing gender and age	Post-adolescent males	Middle-aged females
Development of osteoarthritis	Frequent	Rare
Pathomechanics	Sliding friction (shear contact stress)	Rim-to-neck impact (normal contact stress)
Pathobiology	Inflammation	Scarcely relevant
Pathoanatomy	Primary cam: physeal scar hypertrophy or subclinical SCFE Secondary cam^a^: malunion of femoral neck fracture; SCFE; Legg–Calvè–Perthes disease; femoral head and neck neoplasms; malrotation of the femoral epiphysis; *coxa vara*	Primary pincer: *Protrusio acetabuli* (global overcoverage); acetabular retroversion (anterolateral overcoverage) Secondary pincer: acetabular retroversion after periacetabular osteotomy

*SCFE* slipped capital femoral epiphysis

^a^Theoretically, all the secondary cam deformities might be classified alternatively as femur-based pincer deformities, depending on the likelihood of articular penetration. If the lesion may intrude easily into the joint, it will behave like a cam; otherwise, it will behave like a pincer

## FAI morphology, FAI syndrome and FAI-related hip osteoarthritis

“FAI morphology” is a deformity (or a combination of deformities) that may be detected through an X-ray examination or dedicated CT/MRI, but only seldom becomes symptomatic and deserves to be termed “FAI syndrome” [3]. The factors that keep this condition silent are incompletely acknowledged, but the lumbo-pelvi-femoral complex is suspected to play an important role. This multiple joint complex (to which hips belong) might compensate hip sagittal ROM restrictions with coordinated lumbar spine enhanced curvature (lordosis/kyphosis) and pelvic tilt (anterior/posterior): as far as the restriction is functionally compensated, the subject may be asymptomatic despite positive radiological findings [4]. Athletes might have higher functional demands and develop symptoms more frequently and sooner than non-athletes. Nowadays, no evidence supports surgical correction (arthroscopic or open) of asymptomatic FAI morphology, since neither eventual symptoms onset without surgery nor osteoarthritis prevention with surgery have yet been confirmed. However, the panel of experts participating in the Warwick agreement admit that professional athletes might belong to a very high risk category and case-by-case management should be considered [3].

## Cam morphology: α-angle goniometry and cut-off values

**Fig. 1:**
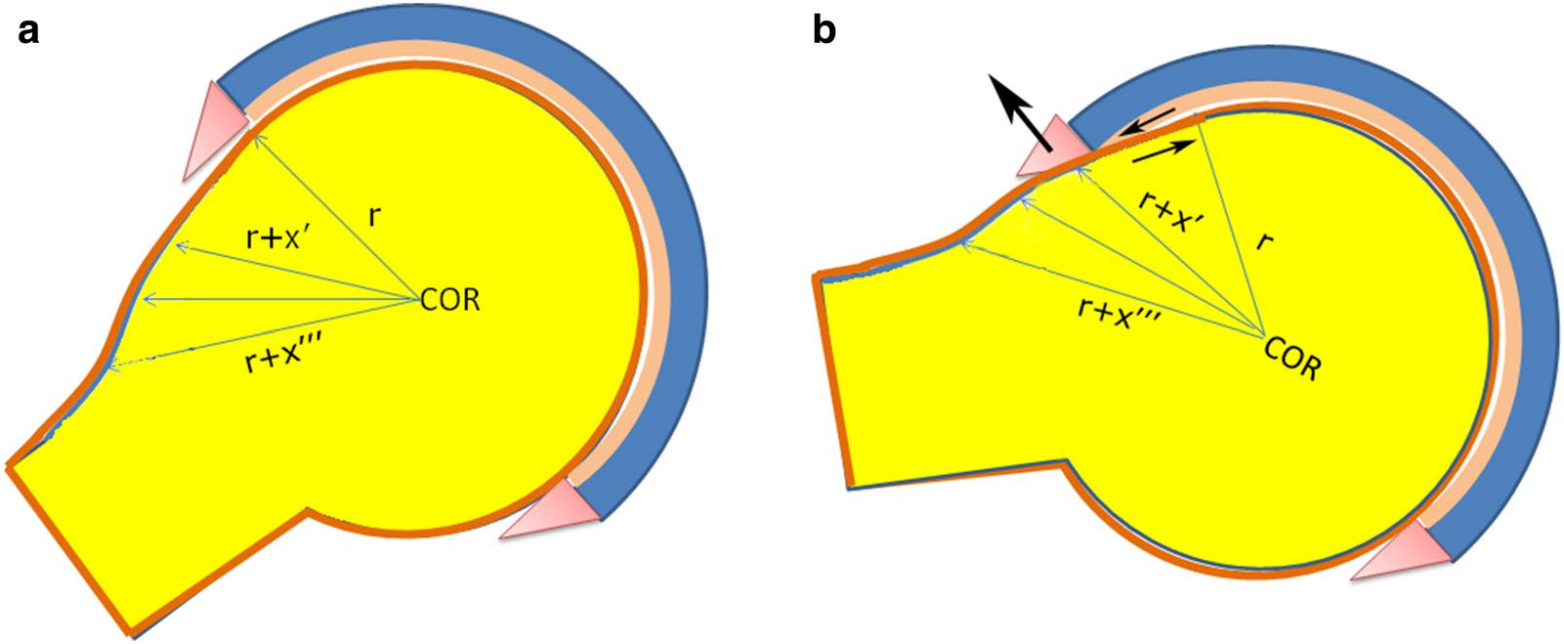
Cam FAI. The cam intrusion is variable, depending on thickness and shape of cam, location, extent, lubrication, force, etc. (*COR* center of rotation, *r* radius, *x* difference between head radius and cam extended radius). **a** Neutral joint position with cam dismorphism in the peripheral compartment; **b** cam intrusion

Cam or “pistol grip” [5] is defined as an aspherical, out-of-round deformity of the femoral head, whose profile shows a growing radius from the proximal border of the lesion to its caudal end (running distally through the head–neck junction and reducing the corresponding head–neck offset) (Fig. 1a, b). It may be associated with a translation or a tilt of the femoral head with respect to the neck axis (e.g., after slipped capital femoral epiphysis, SCFE), but not necessarily. The Nötzli’s α angle is the most popular and comprehensive coordinate describing the location of a cam deformity’s proximal edge [6]: α is the “latitude” of the point where the cephalic radius begins to grow beyond the acetabular radius with a progressive loss of head surface convexity. In this polar reference system the neck axis intersects the best-fit sphere approximating the femoral epiphysis at the 0°-point (virtually caudal pole, located centrally in the head–neck junction) and the neck axis proximal extension intersects the sphere again at the 180°-point (proximal pole). The α-angle characterizing each specific cam deformity is the widest angle among those measured with different plain views of that hip (e.g., anteroposterior view + Dunn 45° and/or cross-table lateral view) perpendicular to the neck or with different 2D reconstructions along the neck if CT or MRI was obtained. To diagnose a cam deformity, α should be at least higher than a cut-off value that is determined by the maximum angular width of a normal neck. A prognostic endpoint (e.g., development of end-stage hip osteoarthritis in the subsequent 5 years) might be added to classify hips according to their fate (normal vs fast deteriorating). Such thresholds depend also on the goniometric method used to measure the α-angle (3-point versus anatomic, as explained below), the statistical method to calculate the cut-off (upper limit of the 95% reference interval for a single population of normally distributed α-angles versus discriminant angular value in case of bimodal distribution of α-angles belonging to two different populations of femoral epiphyses—cam and non-cam), possible prognostic endpoints, ethnicity, gender, sample size, etc. These α-thresholds actually range from 50° [6] to 60° [7].

**Fig. 2:**
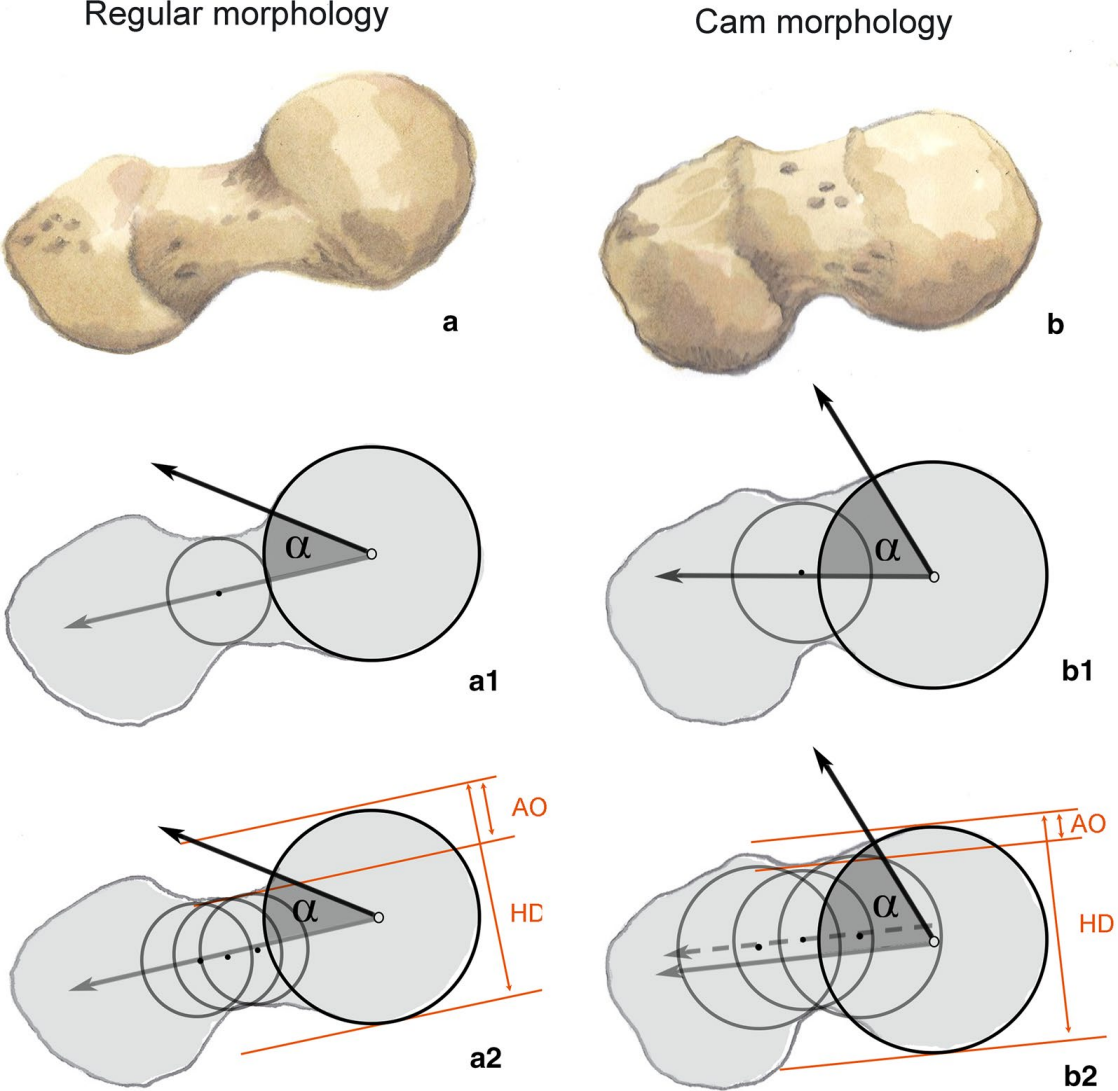
α-Angle goniometry. The proximal femoral epiphysis may exhibit a regular morphology (**a**) or a cam morphology (**b**). Since in **a** the femoral head center is in line with the neck axis, the 3-point method (**a.1**) is as suitable as the anatomic one (**a.2**), and both obtain α = 35° and AOR = AO/HD = 0.217. In **b** the center of rotation of the femoral head is slightly behind the neck axis, thus α = 60° according to the 3-point method (**b.1**, inappropriate), while the anatomic method provides the correct value of 65° (**b.2**). The AOR calculated in **b.2** along the pure mid-neck axis (i.e., disregarding the head position) is 0.108, and belongs to the 2.5% left tail of the normal distribution of AORs (*AOR* anterior offset ratio, *HD* head diameter, *AO* anterior offset)

If head and neck are in line, α may be easily and appropriately determined with a 3-point method (Fig. 2a) by the intersection of 2 straight lines—a first line (“head–neck axis”) joining the center of the best fitting circle around the femoral head and the neck width midpoint at its narrowest section, and a second line joining the above center and the most proximal out-of-round point of the anterior/anterolateral head profile. If head and neck are misaligned, the anatomic method is more suitable (Fig. 2b) than the 3-point one, as it accounts for different anterior and posterior head–neck offsets that otherwise should be provided separately. Here, the first line (termed “mid-neck axis,” as the head position is intentionally ignored) is the best-fit straight line joining at least three width midpoints selected along the neck (whose central one coincides with the midpoint of the minimal neck width of the previous method); the second line stays as previously defined [8]. Unfortunately, the method used for α angle measurement is rarely detailed, which is a remarkable issue as results obtained with the 3-point method are not comparable with those obtained with the anatomic one, at least if the head is not perfectly in line with the neck.

Given that most cam deformities are located on the anterior or anterolateral aspect of the epiphysis just below the equator of the femoral head, many common hip movements (notably flexion, adduction and internal rotation) drive the lesion or part of it into the central compartment of the joint. Moreover, the typical tangential or quasi-tangential cam profile allows thin deformities to slip smoothly below the limbus. Hence cam intrusion is not a rare event, and is suspected to be responsible for the progressive chondral damage on the antero-superior quadrant of the acetabular surface.

The preferential location of the cam explains why lateral or dedicated oblique views (notably the 45° Dunn view [9]) of the proximal femur are more reliable than standard antero-posterior views in detecting cam deformities. A minimal back-shift (< 1 mm) of the head center with respect to the mid-neck axis is extremely common even in normal hips; significant posterior or postero-inferior shift/tilt of the femoral head is often documented in cam FAI, where the anterior head–neck offset, physiologically smaller than the posterior one, is minimal or zero (flat anterior contour) [10]. As anticipated in the previous paragraph, the anterior offset ratio (AOR, or distance between 2 straight lines parallel to the mid-neck axis, tangent to the anterior femoral head and the anterior contour of the thinnest point of the neck, respectively, and normalized for head diameter) should be associated with α angle when the angular measurement method is not anatomic and the lack of convexity is likely underestimated (if the head center is positioned behind the mid-neck axis, the α-angle measured with the 3-point method results in a smaller value). In normal hips mean AOR is 0.19 ± 0.04 and mean overall head-to-neck ratio is 1.75 ± 0.11 [10]. To avoid unnecessary radiation exposure for α measurement and to obtain superior anatomical detail at the same time, MRI and arthro-MRI are progressively replacing multiple plain radiological views. Dedicated radial hip reconstructions allow precise assessment of cam deformities, wherever located, and objective detection of consequent joint injuries.

## Pincer morphology: *coxa profunda* does not mean acetabular overcoverage

*Coxae profundae* were traditionally considered examples of typical pincer morphology with global overcoverage of the femoral head. They are defined as deep sockets where the *fossa acetabuli* is medial to the Kocher’s line and differ from *protrusio acetabuli* because the femoral head is still lateral to this landmark. If the head touches or even protrudes across Kocher’s line, then a true *protrusio* is diagnosed. Recently, no correlation between *coxa profunda* and overcoverage (i.e., LCE or lateral center–edge angle > 40°) could be demonstrated [11]. Thus, many authors discourage considering *coxa profunda* as a radiographic sign of pincer and recommend quantification of the real head coverage by using 2D reconstructions passing through the center of the virtual sphere best fitting the acetabular cavity, and calculating the angle to the center determined by diametrically opposite points of the rim; alternatively, conventional radiological parameters (such as extrusion index, LCE, acetabular arc, acetabular index, Sharp angle, etc. [12]) retain their validity.

## Cam FAI: pathotribology and inflammation pathobiology

Overcoming the simplified rigid-body normal-stress contact mechanics and embracing a more complex but realistic model including shear stresses, viscoelastic bodies and a fluid-lubricated environment, the resulting biotribological perspective may provide additional information [13], useful for in-depth understanding of cam pathomechanics. As a matter of fact, cam intrusion disrupts the fluid film that separates the femoral head and acetabulum, increasing friction. Theoretically, the lubrication regimen of a synovial “ball-and-socket” joint may be boundary (i.e., a monolayer of macromolecules such as lubricin, phospholipids, hyaluronic acid, etc. is linked to each surface, reducing friction even with no fluid interposed) or fluid film-mediated (hydrostatic, or depending on an external device such as a “pump” that pressurizes synovial fluid between joint surfaces; hydrodynamic, where the thick fluid film is gathered by the relative sliding of the articular surfaces, as a function of speed, roughness, curvature, normal load and viscosity; elastohydrodynamic, whose pressurized fluid film is partially determined by the elastic properties of the joint surfaces) or mixed (when the two regimens—boundary and fluid film-mediated—alternate). In the healthy human hip, the acetabular labrum guarantees the pressurization of a supportive film of synovial fluid in the central compartment. Cam intrusion lets synovial fluid escape from the central compartment through the labrum, worsening the lubrication and then favoring wear [14]. For this reason most hip arthroscopists are concerned about limbectomy and aim at preserving the labrum as much as possible, even considering reconstruction with tendon grafts whenever the preservation is not feasible. In a synovial spherical joint model the labrum is everted by cam intrusion and the seal temporarily compromised. Over time, the transition between labral fibrocartilage and hyaline articular cartilage lining the *facies semilunata* is peeled from the subchondral bone (“delamination”) and subsequently torn and worn off. This is how osteoarthritis begins in a mechanistic “wear and tear” scenario. The pathobiological theory of inflammation contributes to support the pathotribological perspective in cam FAI-related osteoarthritis, since fragments of cartilage extracellular matrix released by acetabular wear might trigger and maintain a chronic-recurrent joint inflammation [15, 16].

The fluid film thickness in a synovial joint depends on the following product:

$$\frac{{{\text{Relative}}\;{\text{speed}}\;{\text{of}}\;{\text{joint}}\;{\text{surfaces}}}}{{{\text{Normal}}\;{\text{load}}}} \cdot {\text{viscosity}}\;{\text{of}}\;{\text{synovial}}\;{\text{fluid}} .$$

Inflamed hips are not only poorly lubricated by inflammatory synovial fluid (whose viscosity is decreased because of augmented proteolytic activity), but also slowed down (due to joint pain) and heavily loaded (as joint pain leads to a sedentary lifestyle and often to overweight). All these factors result in increased friction and wear, feeding a vicious loop towards osteoarthritis. Moreover, adipokines released by fat tissue might specifically contribute to joint inflammation [17].

## Pincer FAI and normal-stress contact mechanics

**Fig. 3:**
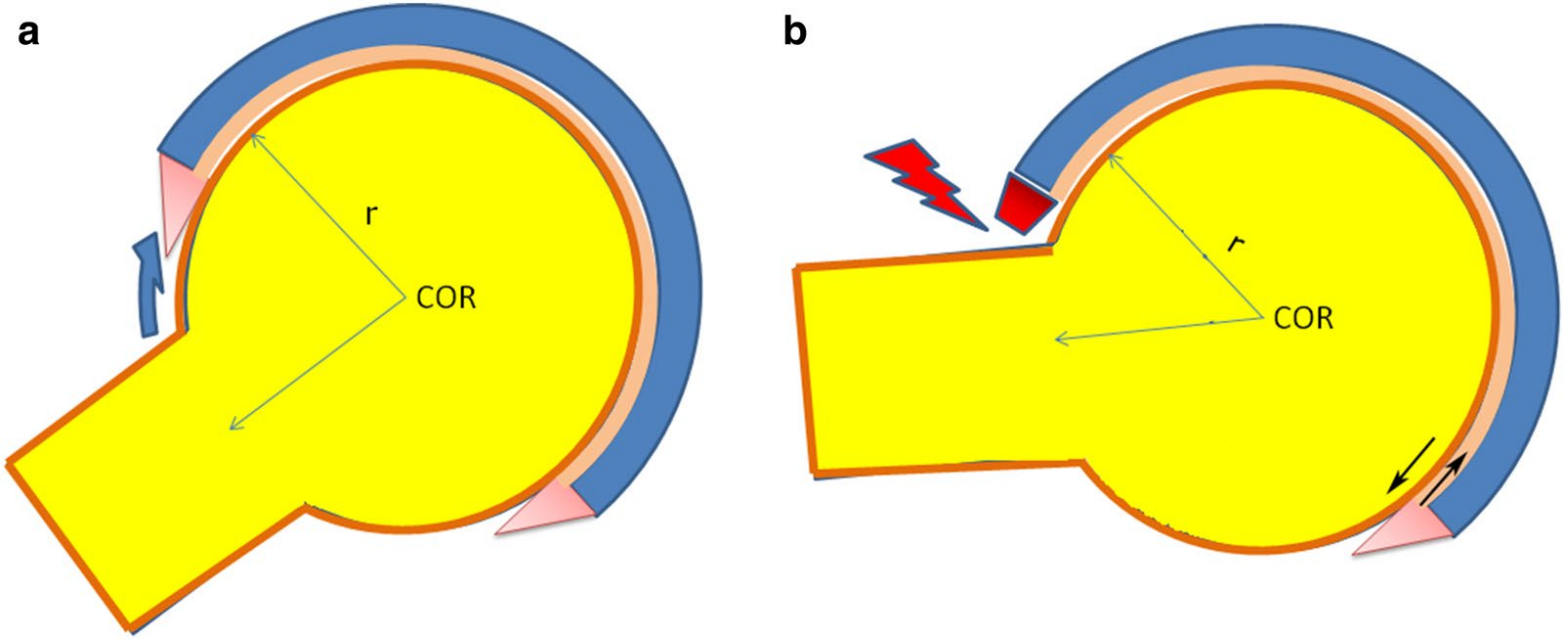
Pincer FAI. The rim-to-neck collision is geometrically determined and represents a firm endpoint for joint range of motion (*COR* center of rotation, *r* radius). **a** Neutral joint position; **b** end range of motion

Pincer FAI occurs when the socket covers the head more than it should and its overhanging rim determines early collisions with the femoral neck, thus limiting the range of motion (ROM). In pincer FAI the impinging structures (or pincers) smash into each other farther from the center of rotation (COR) (Fig. 3a, b) than in cam FAI; the labrum, belonging to the same topographical level as where impingement occurs, gets trapped and squeezed between pincers. Since that is a pure rim-to-neck impact, a normal-stress contact mechanics model is suitable, and the viscoelastic behavior of implied bodies may be the only correction needed. Considering the different contact mechanics of pincer and cam FAI, pincer damages almost exclusively the labrum (i.e., limbus fibrosis with rounded edge, multiple foci of intrasubstance calcification and possible reactive osteogenesis on the back side of the labrum, namely osteophytosis), while cam results frequently in early degenerative joint disease. Pincer FAI subjects could develop early hip osteoarthritis too, because the labral dysfunction affects the pressurization of a supportive synovial fluid film within the joint, but the relative risk is lower and the onset later. On the other hand, it is not a conjecture that if the hip affected by pincer FAI is forcefully pushed beyond the end ROM (e.g., overweight, contact sports, etc.), the site of neck-to-rim collision becomes the fulcrum of a leverage attempting to subluxate the femoral head, with a concentration of shear stresses in the opposite quadrant of the socket (i.e., postero-inferior if impingement occurs antero-superiorly), where occasional chondral injuries may be documented (*contrecoup* lesion).

## Location-pathomechanism mismatch

**Fig. 4:**
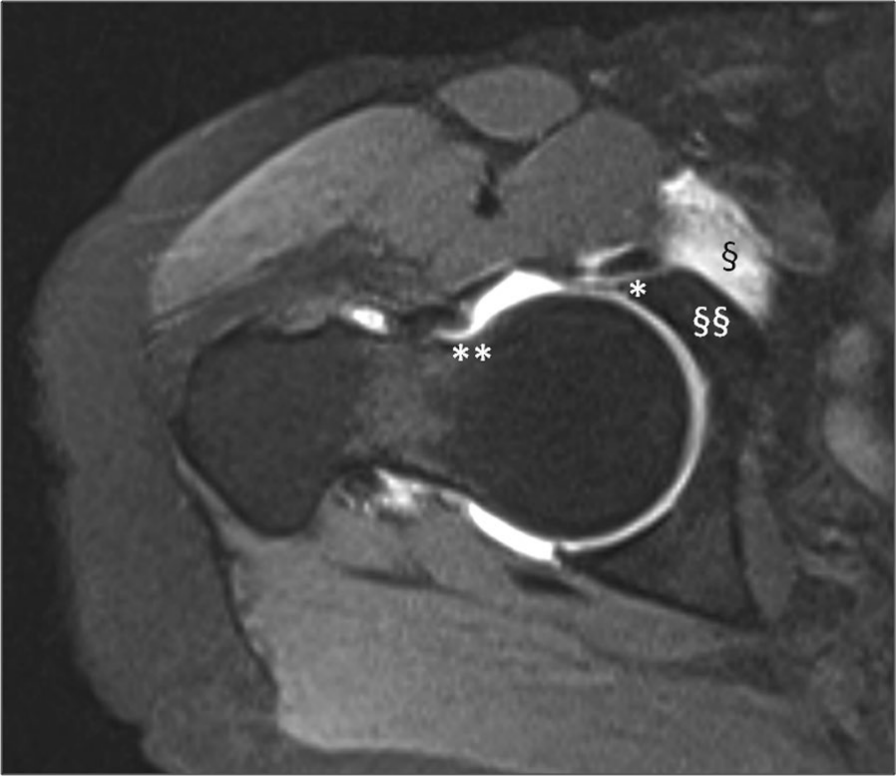
Femur-based pincer FAI morphology. This arthro-MRI axial reconstruction performed with neutral rotation of the limb shows a retroverted neck of femur and some particular features of pincer FAI (*degenerated labrum; ^**^indentation line; ^§^ilio-pectineal *bursa* dilated by contrast medium; ^§§^no signs of cartilage delamination)

Interestingly, although most pincer and cam FAI are respectively caused by acetabular and femoral dysmorphisms, this classification does not depend on dysmorphism location, but on its pathomechanics. Since cam is a deformity of the femoral head–neck surface, cam cannot be but femoral; on the other hand, pincer is determined by the relationship between acetabular opening and femoral neck, thus it may be acetabular (commonly) or femoral (rarely). A poorly oriented neck of the femur (especially in *coxa vara* and *coxa retroversa*) with a normal head–neck junction might induce a pincer-type impingement. This location-pathomechanism mismatch was demonstrated in a case of femoral retroversion due to subtrochanteric derotation osteotomy [18]; if retroversion had occurred at a subcapital level (e.g., due to SCFE), the anterior head–neck offset would have been affected and a conventional cam FAI would have been more likely. Conversely, if a potential cam cannot slip below the limbus because its proximal edge is step-like instead of being smooth, speculatively it might behave like a pincer and represent another example of femur-based pincer FAI, restricting ROM rather than causing true cartilage wear (Fig. 4).

In other words cam and pincer FAI are respectively due to sliding friction and collision, while the location of the deformity, femoral or acetabular, is not pathognomonic. Then occasionally a mismatch may occur (i.e., femur-based pincer FAI).

## Mixed-type FAI

Lastly, cam and pincer may coexist—in this case FAI is defined as “mixed-type” [19] or “combined.” It is likely that such occurrence is rarer than hip arthroscopists report, but cam and pincer are synergic, and a joint affected by a mixed-type FAI should rightly receive prompt and targeted attention as soon as symptoms appear. No wonder the threshold for such a diagnosis is cautiously very low.

## Conclusions

Cam and pincer FAI are quite different issues: both are FAI pathomechanisms, but the former is also a severe pathotribological disorder, dictated by a morphological incongruence of joint surfaces with generation of frictional stress able to seriously injure hip cartilages [20], while the latter preserves the concentric spherical shape of the articular surfaces and behaves as a substantial range of motion restraint with generation of normal stress at sites of rim-to-neck collision [21]. The different contact mechanics make the prediction of ROM more accurate for pincer FAI (where boundaries depend on normal impacts) than for cam FAI (where boundaries depend on maximum cam penetration, which in turn hinges on multiple factors like thickness and shape of cam, location, extent, lubrication, force, etc.). Both mechanisms can be corrected surgically with extensive approaches (surgical dislocation [22] and anterior approach [23]) or minimally invasive techniques (hip arthroscopy [24]), but chances of a persistent satisfactory outcome (i.e., no relapse of symptoms and osteoarthritis prevention) seems to be limited to hips with no or very early arthritic changes, short time elapsed from symptoms onset, and good clinic-functional preoperative performance [25], since chondral status is the weakest link of the chain. Surgical technique, extensive or minimally invasive, has a questionable role, if any. On the other hand, if the condition is asymptomatic, no preemptive surgical correction, neither open nor arthroscopic, is supported by sufficient evidence. In future randomized controlled trials and other high-level-of-evidence studies—some of which are ongoing [26, 27]—might answer these residual big questions (such as what is the natural history of asymptomatic FAI morphology, whether and how we may interfere with it, etc.), that cannot be addressed by simply rearranging the evidence collected so far.

## Data Availability

Not applicable.

## Abbreviations

FAI: femoroacetabular impingement
SCFE: slipped capital femoral epiphysis
AOR: anterior offset ratio
LCE: lateral center–edge angle
ROM: range of motion
COR: center of rotation
